# Supplementary material for: Bidirectional light-emitting diode as a visible light source driven by alternating current
Source: Nat Commun. 2023 Nov 20;14:7562. doi: 10.1038/s41467-023-43335-7 (PMC10661849; doi:10.1038/s41467-023-43335-7)
Supplement: Supplementary file 1 — Supplementary Information [file 41467_2023_43335_MOESM1_ESM.pdf]

Supplementary materials to

Bidirectional light-emitting diode as a visible light source driven by alternating current

Mikołaj Żak[1*], Grzegorz Muziol[1], Marcin Siekacz[1], Artem Bercha[1], Mateusz Hajdel[1], Krzesimir Nowakowski-Szkudlarek[1], Artur Lachowski[1], Mikołaj Chlipała[1], Paweł Wolny[1], Henryk Turski[1], Czesław Skierbiszewski[1]

[1]*Institute of High Pressure Physics Polish Academy of Sciences, Sokołowska 29/37, 01-142 Warsaw, Poland*

**mzak@unipress.waw.pl*

In Fig. S1 we present the calculated tunneling currents as a function of voltage for bottom and top TJ used in examined BD LEDs. They were calculated using a previously developed model that takes into account the spontaneous and piezoelectric charges[1]. In the simulations, the structural parameters of the TJs were set to be identical to those of the top and bottom TJs in the BD LED. As can be seen the calculated tunneling currents are identical for both TJs. The current-voltage characteristics of both TJs is the same, because piezoelectric sheet charges that occur at GaN/$In_{0.02}Ga_{0.98}N$ and $In_{0.02}Ga_{0.98}N$/$In_{0.08}Ga_{0.92}N$ interfaces are located outside of the junction region (see Fig. 2 in main article). Therefore, these charges do not affect tunneling. It will depend only on doping and bandgap, which are identical in top and bottom TJ.

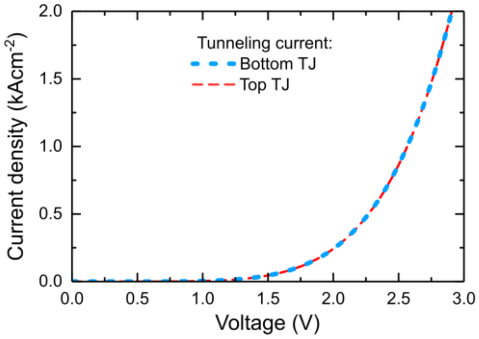

Fig. S1 Calculated tunneling current for bottom and top TJs of BD LED.

In Fig. S2 we present the calculated properties of bidirectional light emitting diode (BD LED) under positive and negative bias. The simulation was performed using 1D-DDCC solver[2-4] at a current density of -1 kAcm^{-2} and 1 kAcm^{-2}, respectively. Figs. S2a and S2b show the schematics of BD LED with applied positive and negative voltage, respectively. Figs. S2c and S2d present the band structure of the active region. The dashed arrow shows the overshoot of electrons due to unfavorable arrangement of the electric field in InGaN quantum well (QW) under positive bias (see Fig. S2c). The difference between the performance of a BD LED at positive and negative bias is significant in the active region. Although the arrangement of the conduction and valence bands and consequently the electric field in the quantum well for both structures are almost the same (see Figs. S2c-d and Figs. S2e-f), it is the crystallographic direction, from which carriers are delivered, that matters. Due to built-in electric fields that are characteristic of growth on Ga-polar GaN substrates, the barrier for electrons in the conduction band on the left-hand side of the QW is significantly higher than on the right-hand side, while in the valence band the barrier for holes is higher on the right-hand side of the QW[5]. Therefore, if electrons are supplied from the right-hand side and holes from the left-hand side, they both face energy barriers preventing carrier escape. The resulting injection efficiency is almost

100% and all the current goes to carrier recombination in the QW. This is the case of negatively biased BD LED. On the other hand, when BD LED is positively biased, the electrons, due to low barrier are prone to escape from the QW. Indeed, this can be observed when analyzing the calculated electron and hole currents, which are presented in Figs. S2g and S2h. We see that in case of positively biased BD LED, part of the electron current overshoots the QW. As expected, in case of biased BD LED, all there is no electron nor hole overshoot and the carriers recombine in the QW. In Figs. S2i and S2j we present carrier concentrations. As can be seen, the two cases, positive and negative biases, do not differ much. The only difference is in the increased hole concentration right above the QW in case of the positive bias (see Fig. S2i).

These band diagram and current flow simulations qualitatively explain the difference in optical power between positive and negative power supply of the BD LED in experiment. In addition, they support the hypothesis of electron overflow, which, we claim, is the main reason for the parasitic peak in electroluminescence that appears at λ=420-430 nm for positively biased BD LED.

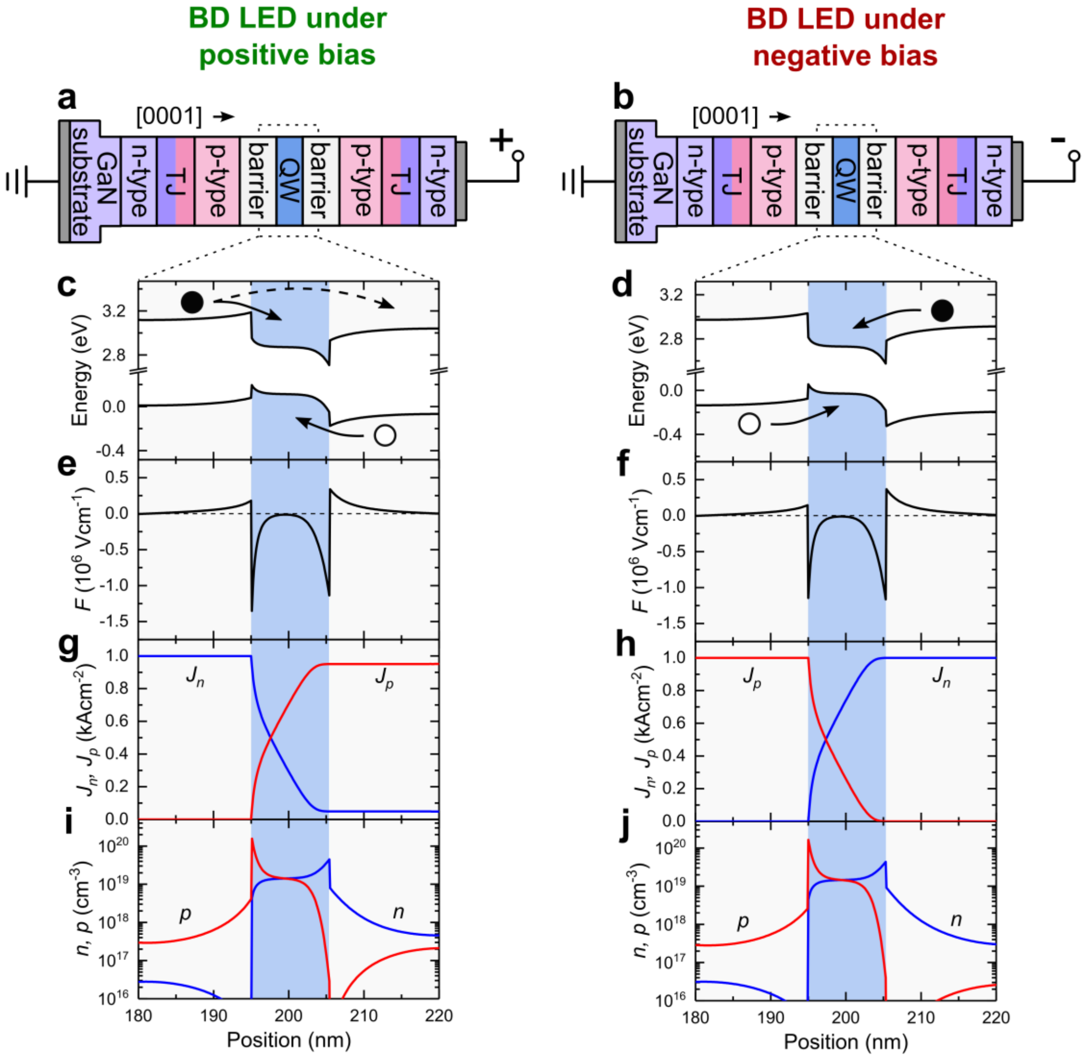

Fig. S2 (a, b) Power supply schemes and (c, d) band diagrams of the BD LED active region under positive (1 kAcm^{-2}) and negative (-1 kAcm^{-2}) biases, respectively. (e, f) Electric fields (F), (g, h) electron (J_n), hole (J_p) current densities and (i, j) electron (n), hole (p) carrier concentration present in the active region of BD

LEDs under positive and negative biases, respectively. Data were obtained by simulation using the 1D-DDCC solver[2-4].

Fig. S3 presents the band diagram of a stack of two BD LEDs fabricated in a single epitaxial process. The carrier flow inside each section from the stack is the same as in a single BD LED. However, in this case, the current flow between each individual BD LEDs in the stack is important. For both positive and negative bias it is only the electron current.

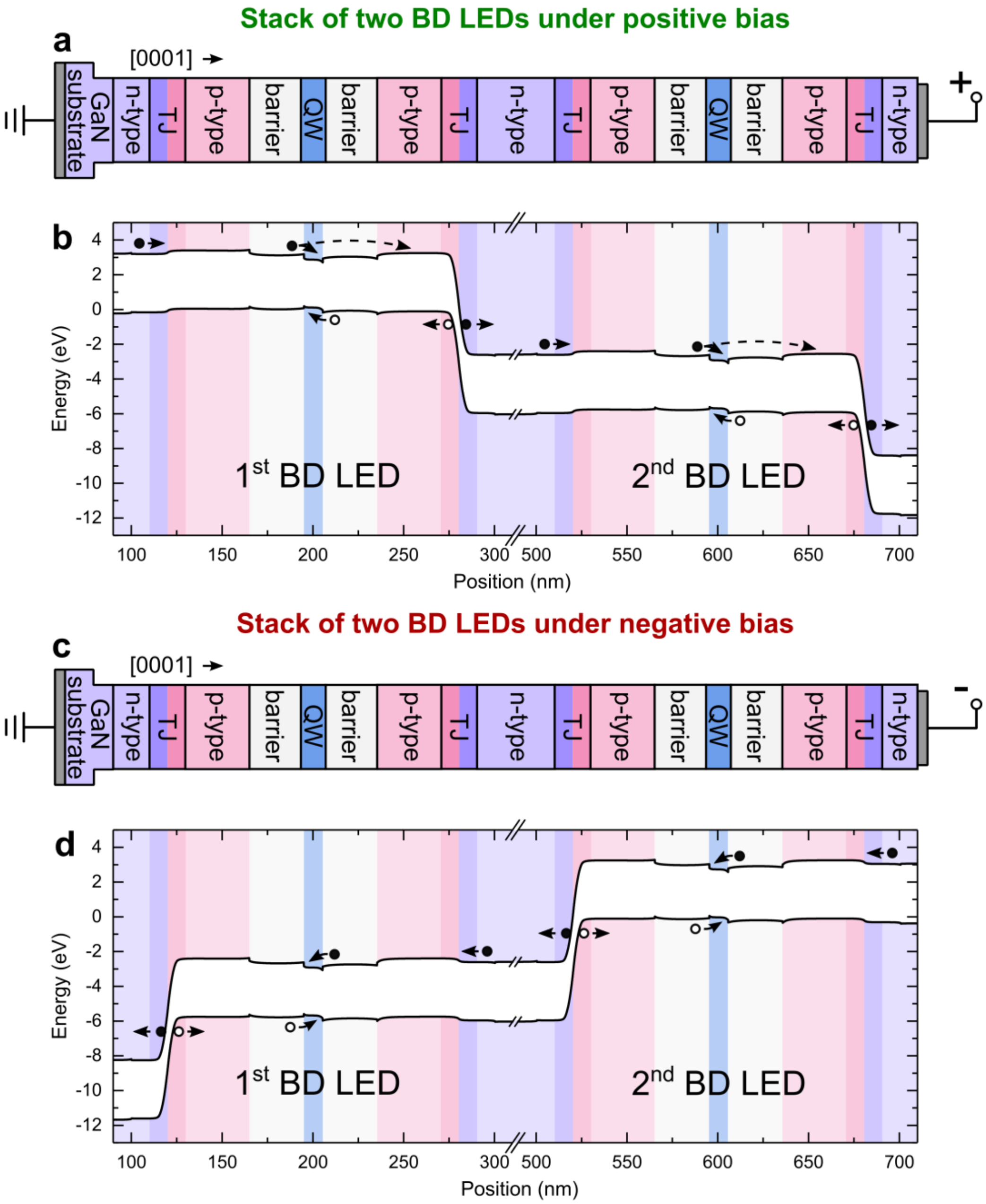

Fig. S3 (a, c) Power supply schemes and (b, d) band diagrams of stacks of two BD LEDs under positive and negative bias regime, respectively, calculated for current density of 1 $kAcm^{-2}$ and -1 $kAcm^{-2}$. Arrows present the direction of electron and hole currents.

Supplementary references:

1. Żak, M. *et al.* Tunnel Junctions with a Doped (In,Ga)N Quantum Well for Vertical Integration of III-Nitride Optoelectronic Devices. *Physical Review Applied* **15**, 024046, doi:10.1103/PhysRevApplied.15.024046 (2021).
2. Wu, Y.-R., Singh, M. & Singh, J. Gate leakage suppression and contact engineering in nitride heterostructures. *Journal of Applied Physics* **94**, 5826-5831, doi:10.1063/1.1618926 (2003).
3. Wu, Y.-R. & Singh, J. Metal piezoelectric semiconductor field effect transistors for piezoelectric strain sensors. *Applied Physics Letters* **85**, 1223-1225, doi:10.1063/1.1784039 (2004).
4. Wu, Y. R., Chiu, C., Chang, C. Y., Yu, P. & Kuo, H. C. Size-Dependent Strain Relaxation and Optical Characteristics of InGaN/GaN Nanorod LEDs. *IEEE Journal of Selected Topics in Quantum Electronics* **15**, 1226-1233, doi:10.1109/JSTQE.2009.2015583 (2009).
5. Turski, H., Bharadwaj, S., Xing, H. & Jena, D. Polarization control in nitride quantum well light emitters enabled by bottom tunnel-junctions. *Journal of Applied Physics* **125**, 203104, doi:10.1063/1.5088041 (2019).
